# Tinea Capitis Caused by *Microsporum audouninii*: A Report of Two Cases from Côte D’Ivoire, West Africa

**DOI:** 10.3390/tropicalmed6010009

**Published:** 2021-01-12

**Authors:** Rie Roselyne Yotsu, Kouamé Kouadio, Aubin Yao, Bamba Vagamon, Motoi Takenaka, Hiroyuki Murota, Koichi Makimura, Katsutaro Nishimoto

**Affiliations:** 1 School of Tropical Medicine and Global Health, Nagasaki University, Nagasaki 852-8523, Japan; 2 Department of Dermatology, National Center for Global Health and Medicine, Tokyo 162-8655, Japan; 3 Department of Tropical Medicine, Tulane University School of Public Health and Tropical Medicine, New Orleans, LA 70118, USA; 4 Eco Epidemiology Unit, Pasteur Institute of Côte d’Ivoire, Abidjan, Cote D’Ivoire; kouadiokouame@yahoo.com; 5 Hope Commission International, Abidjan, Cote D’Ivoire; aubin@hopecommission.org; 6 Raoul Follereau Institute Côte d’Ivoire, Adzopé, Cote D’Ivoire; bambavagamon@yahoo.com; 7 Department of Dermatology, Université Alassane Ouattara, Bouaké, Cote D’Ivoire; 8 Department of Dermatology, Nagasaki University Graduate School of Biomedical Sciences, Nagasaki 852-8501, Japan; m-take@nagasaki-u.ac.jp (M.T.); h-murota@nagasaki-u.ac.jp (H.M.); 9 Medical Mycology, Graduate School of Medicine, Teikyo University, Tokyo 173-8605, Japan; makimura@med.teikyo-u.ac.jp; 10 Nagasaki Ekisaikai Hospital, Nagasaki 850-0034, Japan; kjwest@ekisaikai-nagasaki.jp

**Keywords:** dermatophytosis, dermatophyte, developing country, *Microsporum audouinii*, sub-Saharan Africa, tape sampling, tinea, tinea capitis

## Abstract

We report here two cases of tinea capitis caused by *Microsporum (M.) audouinii* in Côte d’Ivoire, West Africa. The patients were a three-year-old boy and a six-year-old girl who presented with scaly patches on the scalp. The causative fungus was isolated using an adhesive tape-sampling method and cultured on Sabouraud dextrose agar plates. It was identified as *M. audouinii* both by its macroscopic and microscopic features, confirmed by DNA sequencing. These are the first documented cases of *M. audouinii* infections confirmed with DNA sequencing to be reported from Côte d’Ivoire. The practicality of the tape-sampling method makes it possible to carry out epidemiological surveys evaluating the distribution of these dermatophytic infections in remote, resource-limited settings.

## 1. Introduction

Tinea capitis is an infection of the scalp due to keratinophilic fungi, also known as dermatophytes. They are filamentous fungi that digest and grow on keratinized tissues including skin, hair, and nails. It is most prevalent in children in developing countries, affecting mostly persons of low socio-economic status [1,2,3,4]. However, the reported prevalence is likely an underestimation as the conditions are often neglected or ignored due to the non-fatal nature compared to other health issues prevalent in settings where they are endemic [5]. There is also a paucity of laboratories capable of performing mycological examinations, so diagnostic confirmation is rarely done [6]. The causative organisms vary by geographic setting [4,6], and only a few studies on tinea capitis with laboratory confirmation have been conducted in sub-Saharan African countries [7,8]. We report here the first two cases of tinea capitis by *Microsporum* (*M.*) *audouinii* in Côte d’Ivoire confirmed by culture and DNA sequencing. 

## 2. Materials and Methods

### 2.1. Skin Surveys

We conducted a cross-sectional skin survey in the Oumé Health District of Côte d’Ivoire from October 21 to November 30, 2019 as described previously [9]. Oumé is located approximately 200 km north of Abidjan, the country’s economic capital, with a population of 274,020 (2018 est.) who are mostly farmers. The main purpose of our surveys was to identify neglected tropical diseases (NTDs) with skin manifestations (so-called “skin-NTDs”, including leprosy, Buruli ulcer, and yaws) that are co-endemic in the study site. We first conducted a sensitization campaign informing the community members of the availability of skin disease specialists who will conduct free consultations of the children. The consultations were conducted at the local healthcare center or at classrooms in the primary school, if privacy could be secured.

We pilot-tested the specimen collection—use of transparent adhesive tape on the scalp—on 10 cases with clinical diagnosis of tinea capitis infection, as described below.

### 2.2. Laboratory Investigations

Samples for the mycological examination were collected by a piece of transparent adhesive tape of approximately 5 cm in length. The adhesive side of the tape was pressed firmly on the surface of the scalp lesions to be examined. After removal, they were placed between two glass slides, and sent to the mycology laboratory in the Department of Dermatology, Nagasaki University Hospital. There, the tape was placed on the surface of Sabouraud dextrose agar (SDA) supplemented with chloramphenicol and cycloheximide, and was incubated at room temperature. The slides were visually inspected once weekly for four weeks for evidence of growth. Observed colonies were sub-cultured and the positive cultures were sent to the Medical Mycological Laboratory, Teikyo University, for DNA sequencing. Briefly, DNA from the fungal cells was rapidly extracted and the DNA fragments covering the nuclear internal transcribed spacer (ITS) region of ribosomal RNA gene were amplified with two primers (ITS1 and ITS4) as described previously [10,11]. The PCR products were directly sequenced with these primers. 

### 2.3. Ethical Declaration

The study was evaluated and approved by the Life Sciences and Health of Côte d’Ivoire (N/Réf:078-19/MSHP/CNESVS-kp) and the Ethics Committee of the School of Tropical Medicine and Global Health of Nagasaki University (NU_TMGH_2019-097-0). Written informed consent and assents were obtained from the study participants and their guardians.

## 3. Case Reports and Results

**Figure 1:**
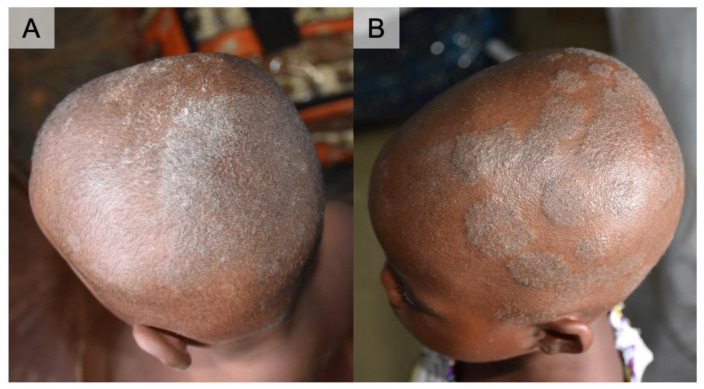
Clinical presentation of two cases of *Microsporum audouinii.* (**A**) Case 1 in a three-year-old boy. Irregular patches of whitish to silver scales with breaking hairs of the scalp. (**B**) Case 2 in a six-year-old girl. Raised and well-defined multiple patches of the scalp. She had her hair shaved as part of the treatment (local practice).

A three-year-old boy (Case 1) and six-year-old girl (Case 2) both presented with multiple scaly patches with silvery-white scales on the scalp and breaking hairs forming patches of partial alopecia (Figure 1). The patches were especially raised and well-defined in Case 2. Clinical diagnosis of tinea capitis was made on site, and oral griseofulvin (dosage calculated based on body weight) and topical ketoconazole were given to the patients along with parental education. Theirs were the two positive cultures out of the 10 tape samples that were obtained during the survey. 

**Figure 2:**
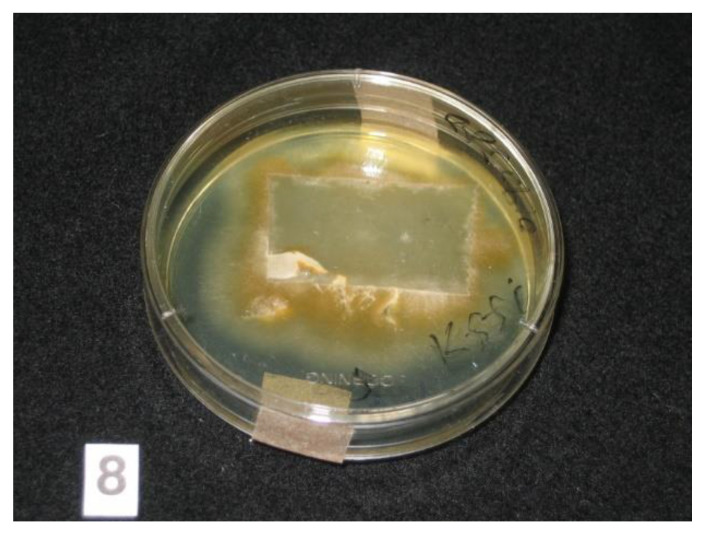
Adhesive tape sample containing scales and hair placed on Sabouraud dextrose agar plate. Growth of colonies of dermatophyte was seen from the edges of the tape at Week 3.

**Figure 3:**
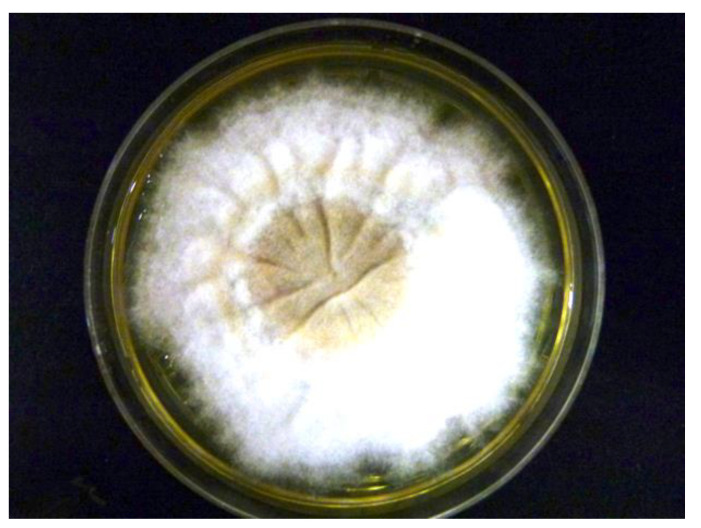
Macroscopic morphological features of *Microsporum audouinii* grown on Sabouraud dextrose agar plate at Week 3. Flat, velvety, whitish colonies were cultured.

**Figure 4:**
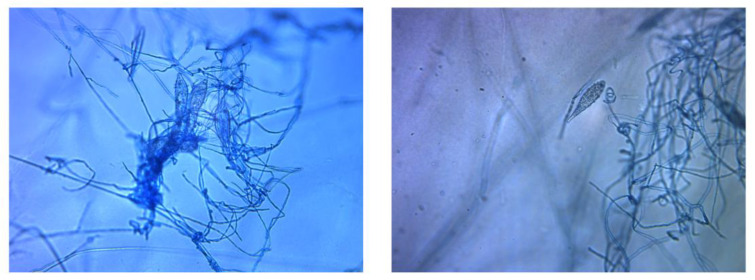
Microscopic morphological features of *Microsporum audouinii.* Spindle-shaped, septate macroconidia with echinulate surface (magnification x200).

**Figure 5:**
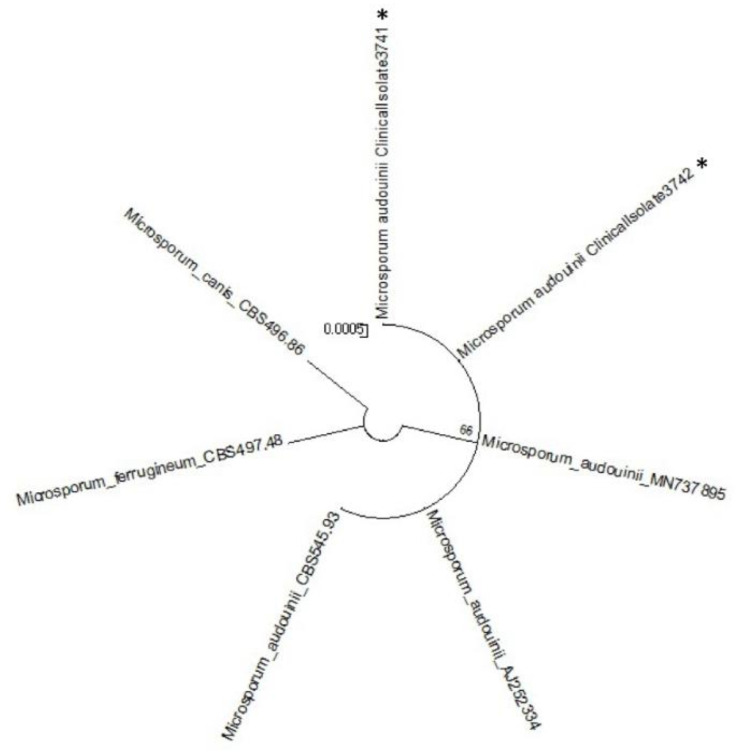
Molecular phylogenetic tree of the two strains of *Microsporum audouinii* from our study (shown with ⁎).

Adhesive tapes were placed on the SDA plates and colonies of dermatophyte were observed from the edges of the tapes at week 3 (Figure 2). Upon sub-culture, flat, velvety whitish colonies were observed (Figure 3). Microscopic examination of the colonies showed spindle-shaped, echinulate macroconidia with 5 to 8 septa (Figure 4). Morphological and physiological features were compatible with *M. audouninii*. This was further confirmed by DNA sequencing which showed 100% (320/320) homology to the *M. audouninii* species using the MycoBank BLAST program (accession number: AJ252334) [12,13] (Figure 5).

## 4. Discussion

We hereby present two cases of tinea capitis with microbiologic confirmation of *M. audouninii* infection from Côte d’Ivoire. 

Dermatophytoses are the most frequent forms of fungal infections, affecting 20−25% of the human population [3,14]. Tinea capitis predominantly affects children and is highly prevalent in sub-Saharan Africa [2].

A number of skin surveys aimed at detecting skin NTDs have revealed a high prevalence of dermatophytoses, including tinea capitis in NTD-endemic regions of sub-Saharan Africa [9,15,16], with our previous survey in another district of Côte d’Ivoire showing an 11% prevalence of tinea capitis. However, confirmatory diagnosis is not performed in most instances, and the causative organisms are not identified given the lack of laboratory facilities. Therefore the true prevalence of tinea capitis, the geographic distribution, and the most common causative organisms remain unclear. In one report, *M. audouinii* was one of the most prevalent species in schoolchildren with tinea capitis in Gabon (25%) following *Trichophyton (T.) soudanense* (29%) and *T. tonsurans* (28%) [1,4]. Two reports from Côte d’Ivoire also reported *T. soudanense* and *M. audouinii* as the two predominant species on microscopy [17,18]. Ours are the first reports of *M. auduinii* causing tinea capitis in Côte d’Ivoire, confirmed by culture and DNA sequencing. 

*M. audouinii* is commonly regarded as an anthropophilic dermatophyte since isolation from animals and soil is rare [19,20]. Transmission is thought to be human-to-human through habitual contact [21]. A number of risk factors for acquiring tinea capitis have been reported, including ethno-cultural factors such as hairdressing mode, socio-economic factors, and climatic factors such as humidity [22,23,24]. Causative organisms of tinea capitis vary by region. *M. audouinii* is predominantly isolated in Africa but is increasingly reported in Europe and the Americas likely as a result of population migrations [6,25,26,27,28,29]. This indicates why there is a need for continuous surveillance not only to better determine the prevalence but also for the changes in causative species of dermatophytosis. 

Clinical manifestations of tinea capitis can vary by the extent of inflammation that it causes. Infection by *M. audouinii* is known to be associated with no or mild inflammatory response. It commonly starts as a small erythematous papule surrounding a single hair shaft, which gradually spreads centrifugally to surrounding follicles leading to patches of alopecia. These are usually circular in shape with numerous broken-off hairs as well as fine scaling, which characteristic features also observed in our cases [1,14]. Unlike *M. canis* or *M. gypseum* infections, there is usually no pruritus [1,14], which might in part account for the lack of requests for medical attention [5], and likelihood of spreading the disease from untreated cases. 

We have successfully isolated the organisms using the adhesive tape-sampling method. This is a quick, non-invasive technique (as compared to scalpel blade sampling) and very amenable to use in field conditions. Its sensitivity has been reported to be at least comparable to that of the scalpel blade sampling reporting over 90% [30,31]. It was also very acceptable and non-threatening to children leading to a high rate of adherence [30,31]. In addition, collected specimens could be more easily transported and stored for a longer time as was the case in our study [31,32]. The combination of increased awareness, field-adapted methods like tape sampling, and greater availability of sophisticated technologies like DNA sequencing for species identification will soon allow a much better understanding of the prevalence and distribution of dermatophytic infections.

Our future plan is to build the capacity within the country of Côte d’Ivoire by training technicians and obtaining equipment to perform the same laboratory analyses in a timely manner in order to achieve that goal. 

## 5. Conclusions

The presence of *M. audouinii* was identified as a causative species of tinea capitis in two children diagnosed during a skin survey conducted in rural Côte d’Ivoire through culture and DNA sequencing. The tape-sampling method was found to be an effective method in field settings for collecting samples from affected children. Optimization of mycology diagnostic methods and capacities in epidemiological studies will support in providing more understanding of the prevalence and distribution of this neglected but highly prevalent skin condition in sub-Saharan African countries. 

## Data Availability

Data is contained within the article.

## Abbreviations

The following abbreviations are used in this manuscript:

*M.*	*Microsporum*
PCR	polymerase chain reaction
SDA	Sabouraud dextrose agar
